# Telomerase reverse transcriptase acts in a feedback loop with NF-κB pathway to regulate macrophage polarization in alcoholic liver disease

**DOI:** 10.1038/srep18685

**Published:** 2016-01-04

**Authors:** Xiao-qin Wu, Yang Yang, Wan-xia Li, Ya-hui Cheng, Xiao-feng Li, Cheng Huang, Xiao-ming Meng, Bao-ming Wu, Xin-hua Liu, Lei Zhang, Xiong-wen Lv, Jun Li

**Affiliations:** 1 School of Pharmacy, Anhui Key Laboratory of Bioactivity of Natural Products, Anhui Medical University, Hefei 230032, China; 2 The Key Laboratory of Anti-inflammatory and Immune Medicine, Anhui Medical University, Ministry of Education, Hefei 230032, China; 3 Institute for Liver Diseases of Anhui Medical University, ILD-AMU, Anhui Medical University, Hefei 230032, China; 4 Anhui Institute of Innovative Drugs, Anhui Medical University, Hefei 230032, China

## Abstract

Activation of Kupffer cells (KCs) plays a central role in the pathogenesis of alcoholic liver disease (ALD). C57BL/6 mice fed EtOH-containing diet showed a mixed induction of hepatic classical (M1) and alternative (M2) macrophage markers. Since telomerase activation occurs at critical stages of myeloid and lymphoid cell activation, we herein investigated the role of telomerase reverse transcriptase (TERT), the determining factor of telomerase, in macrophage activation during ALD. In our study, TERT expression and telomerase activity (TA) were remarkably increased in liver tissue of EtOH-fed mice. Moreover, EtOH significantly up-regulated TERT in isolated KCs and RAW 264.7 cells and LPS induced TERT production *in vitro*. These data indicate that up-regulation of TERT may play a critical role in macrophages during ALD. Furthermore, loss- and gain-of-function studies suggested that TERT switched macrophages towards M1 phenotype by regulating NF-κB signaling, but had limited effect on M2 macrophages polarization *in vitro*. Additionally, PDTC, a chemical inhibitor of NF-κB, could dramatically down-regulate TERT expression and the hallmarks of M1 macrophages. Therefore, our study unveils the role of TERT in macrophage polarization and the cross-talk between TERT and p65, which may provide a possible explanation for the ethanol-mediated hepatic proinflammatory response and M1 macrophage polarization.

Alcoholic liver disease (ALD) is one of the predominant causes of liver-related morbidity and mortality worldwide^1^,^2^ and is the basis of approximately 50% of cirrhosis cases in the United States^3^. Recently, because of a noteworthy increase in alcohol consumption in Asia, ALD is becoming a leading cause of chronic liver disease as well^1^. The spectrum of ALD includes steatosis, steatohepatitis, alcoholic fibrosis, cirrhosis and even hepatocellular carcinoma (HCC)^4^. So far, several pathogenic mechanisms of ALD, including the interaction between direct toxic effects of alcohol and its metabolites in target cells, has been identified^5^. Interestingly, alcohol metabolism and increased toxic reactive metabolites and oxidative stress play vital roles in the initiation of ALD^6^,^7^. Accumulating data indicate that subsequent activation of the innate immune cell and circulating endotoxin/lipopolysaccharide (LPS) are a crucial aspect of the potential mechanism^8^. Circulating endotoxin/LPS activates hepatic macrophages (kupffer cells, KCs) and then induces numerous cytokines, chemokines, and reactive oxygen species^9^,^10^. An increased number of macrophages have been found in different stages of ALD^11^. Nevertheless, the functions of KCs in the pathogenesis of ALD have not been fully addressed.

It has been demonstrated the considerable plasticity of macrophages, which alter their phenotype and physiology in response to various cytokines and microbial signals^12^. On the basis of the TH1 (T helper type 1)-TH2 polarization idea^13^, Macrophages may undergo classical M1 activation, or alternative M2 activation. M1 macrophages are stimulated by Toll-like receptors (TLRs) ligands and/or interferon-γ (IFN-γ) and characterized by high levels of pro-inflammatory cytokines, such as interleukin (IL)-1β and tumor necrosis factor-α (TNF-α), with high production of reactive nitrogen and oxygen intermediates. M1 macrophages mediate resistance to pathogens and contribute to tissue damage, while M2 macrophages are treated with IL-4 or IL-13 and produce anti-inflammatory cytokines including IL-10, as well as promote tissue remodeling and tumor progression^14^,^15^,^16^. Although such dichotomy is useful, the M1/M2 phenotypes of macrophages are oversimplified, attributing to the substantial plasticity of macrophages *in vivo*^17^. Macrophages have a unique ability to switch their phenotypes based on the tissue microenvironmental cues, such as growth factors, cytokines and pathogen-associated molecular pattern (PAMP) molecules^18^. The heterogeneity of macrophages is reflected on their differential, sometimes even opposing, roles in kinds of diseases^19^,^20^,^21^. The characterization of macrophage phenotypes provides important tools for revealing the regulation of inflammatory processes in the progression of ALD. It has been found that M2 KCs can promote hepatocyte senescence and M1 KCs apoptosis to protect against ALD^22^,^23^. However, the regulatory factors that underlie macrophage polarization remain largely undefined. Hence, it is extremely important to understand the potential molecular mechanisms underlying the control of macrophage polarization during ALD.

Telomerase is a ribonucleoprotein (RNP) composed of two major components: telomerase RNA (TR) and telomerase reverse transcriptase (TERT). TR is universally detected in an abundance of cells and acts as a template for TERT; whereas TERT is expressed in low levels in most adult cells and catalyzes the enzymatic reaction of DNA synthesis^24^. Although most normal cells display low basal telomerase activity (TA), emerging evidence suggest that TERT is tightly regulated in its expression and required to reactivate telomerase in telomerase-negative cells^25^. Telomerase reactivation has been demonstrated in many pathological processes, mainly in carcinomas^26^ but also in inflammatory disorders^27^ and liver diseases^28^. Recently, it has been reported that TERT expression is induced and telomerase is reactivated in macrophages after inflammatory stimuli including LPS, oxidized low-density lipoprotein (oxLDL) and TNF-α. More importantly, this induction of TERT expression may have major implications for the development of atherosclerosis^29^. These studies support the idea that TERT can be activated in macrophages in the presence of specific stimuli that may correspond to immune responses. Given the dramatic increase of TERT expression and TA upon inflammatory stimuli, we hypothesized that TERT might be involved in alcohol-induced macrophage polarization in ALD. In this study, we sought to define the potential roles of TERT in macrophage polarization and the molecular mechanisms underlying this regulation in ALD.

## Results

### Pathological characteristics of ALD and characterization of the phenotypes of KCs in mice after chronic and binge ethanol feeding

**Figure 1:**
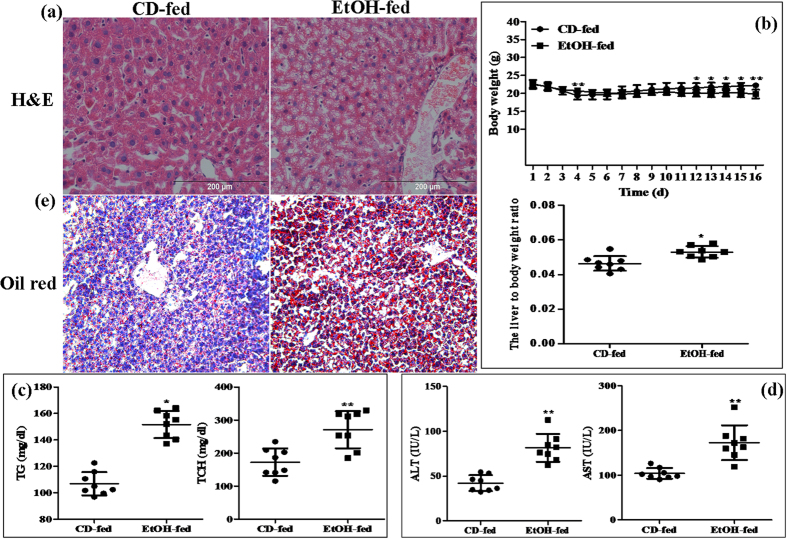
Pathological characteristics in a mouse model of alcoholic liver disease (ALD). (**a**) Representative hematoxylin and eosin (H&E) staining of liver tissues. (**b**) Body weights and the liver to body weight ratio after ethanol feeding. (**c**) Hepatic triglyceride (TG) and total cholesterol (TCH) levels. (**d**) Serum ALT and AST levels. (**E**) Representative Oil Red O staining of liver tissues. The values represent means ± SD. (n = 8 in CD-fed group, n = 8 in EtOH-fed group) *P < 0.05, **P < 0.01 *vs* CD-fed group.

All of male C57BL/6J mice with chronic alcohol feeding were characterized by immune cell activation, inflammation, injury and steatosis in the liver. Firstly, the degree of liver injury was evaluated in all EtOH-fed mice by hematoxylin eosin (H&E) staining. Histopathological analysis showed that degree of alcohol-induced liver injury progressed in EtOH-fed mice relative to the control diet (CD)-fed mice. Liver tissues in EtOH-fed mice exhibited fat vacuoles, liver cell cord derangement, intercellular spaces dilatation and inflammatory cell infiltration, while CD-fed mice showed normal lobular architecture with central veins and radiating hepatic cords (Fig. 1a). Furthermore, the body weights of both EtOH-fed mice and CD-fed mice were gradually decreased in the beginning, and then slightly increased after the adaptive phase. Interestingly, body weights of EtOH-fed mice were significantly lower than those of the control group, while the liver to body weight ratio in the EtOH group was remarkably higher than that in the control group at the end of model building (Fig. 1b). To determine the effect of EtOH ingestion on lipid homeostasis, hepatic steatosis developed in the model of ALD was assessed. As shown in Fig. 1c, there was a dramatic increase in serum TG and TCH levels in EtOH-fed mice. Moreover, these metabolic changes were associated with a significant increase in serum levels of ALT and AST (Fig. 1d), suggesting that EtOH consumption induced hepatocellular injury in this mouse model. In line with the above data, the number of lipid droplet was significantly increased in liver tissue of EtOH-fed mice compared to the CD-fed mice (Fig. 1e).

**Figure 2:**
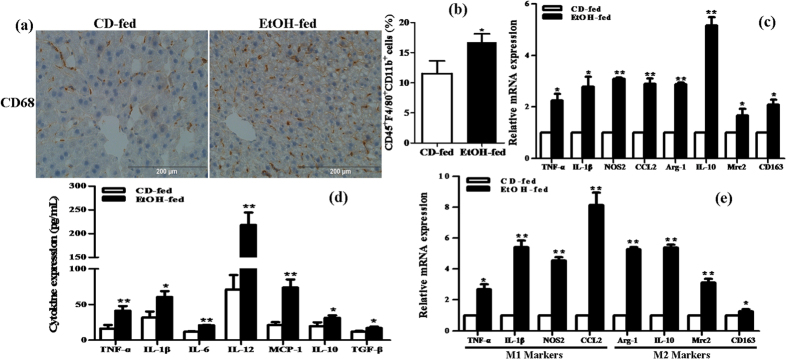
Characteristic of the liver immune cell population in the progression of ALD. (**a**) The expression of CD68 in liver tissue was analyzed by immunohistochemical (IHC) staining analysis. Representative views from each group were presented (original magnification, ×40). (**b**) Effect of alcohol on the number of CD45^+^F4/80^+^CD11b^+^ cells in KCs isolated from the liver by using FACS analysis. (**c**) Effect of alcohol on the mRNA levels of M1 macrophage markers (TNF-α, IL-1β, IL-6 and CCL2) and the M2 macrophage marker including Arg-1, IL-10, Mrc2 and CD163 in liver tissue. (**d**) Effect of alcohol on the circulation levels of pro-inflammatory cytokines (TNF-α, IL-1β, IL-6, IL-12 and MCP-1) and anti-inflammatory cytokines (IL-10 and TGF-β) in serum. (**e**) Effect of alcohol on the mRNA levels of M1/M2 macrophage markers in KCs isolated from the liver. The results are shown as relative expression against control expression without treatment. The values represent means ± SD. (n = 4 in CD-fed group, n = 8 in EtOH-fed group) *P < 0.05, **P < 0.01 *vs* CD-fed group.

Next, we investigated the characteristic of the liver immune cell population in the progression of ALD. Immunohistochemical (IHC) staining analysis showed that more CD68^+^ cells in liver tissue were detected in the EtOH group compared to the control group (Fig. 2a). In FACS analysis of KCs isolated from the liver, there was an approximate 1-fold increase in the frequency of CD45^+^F4/80^+^CD11b^+^ cells in the EtOH group compared to the control group (Fig. 2b). Furthermore, liver tissues in EtOH-fed mice showed a significant increase in the mRNA levels of TNF-α, IL-1β, IL-6, CCL2, Arg-1, IL-10, Mrc2 and CD163 compared with CD-fed mice (Fig. 2c). We further evaluated the circulation levels of inflammatory cytokines in serum from EtOH-fed mice. ELISA results showed that the serum levels of various proinflammatory cytokines such as TNF-α, IL-1β, IL-6, IL-12 and MCP-1 were notably elevated, and the levels of anti-inflammatory cytokines including IL-10 and TGF-β were also observably increased in the EtOH group (Fig. 2d). In addition, we isolated KCs from the liver of EtOH-fed mice and CD-fed mice and also measured the levels of M1/2 macrophage markers *in vivo*. M1 macrophage markers TNF-α, IL-1β, NOS2 and CCL2 were remarkably up-regulated in the EtOH group. Similarly, there was substantial difference in the level of the M2 macrophage markers Arg-1, IL-10, Mrc2 and CD163 between the two groups (Fig. 2e). Therefore, all above data suggests that chronic and binge ethanol feeding induces a mixed M1/M2 macrophage phenotype *in vivo*.

### EtOH-fed mice have higher levels of TERT than CD-fed mice

**Figure 3:**
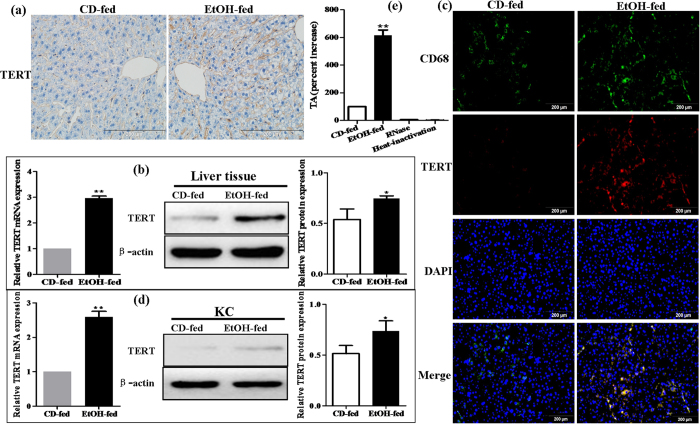
Effect of alcohol on TERT expression in liver tissues and KCs during ALD development. (**a**) TERT expression in liver tissues was performed by IHC analysis. Representative views from each group were presented (original magnification, ×40). (**b**) Total TERT mRNA and protein levels in liver tissue were analyzed by real-time PCR and western blot. The results are shown as relative expression against control expression without treatment. (**c**) Representative colocalization of TERT with macrophage CD68 immunoreactivity in liver tissue by using the double immunofluorescent (IF) analysis. (**d**) Total TERT mRNA and protein levels in KCs isolated from the liver were analyzed by real-time PCR and western blot. The results are shown as relative expression against control expression without treatment. (**e**) Quantification of telomerase activity (TA) in CD-fed mice and EtOH-fed mice. RNase treatment or heat inactivation of KCs isolated from the liver of EtOH-fed mice served as negative controls for the TA assay. All quantitative data are presented as mean ± SD percentage increase compared with CD-fed group (n = 4 in CD-fed group, n = 6 in EtOH-fed group) *P < 0.05, **P < 0.01 *vs* CD-fed group.

To identify the changes in the TERT expression profile between EtOH-fed mice and CD-fed mice, IHC analysis and western blot on liver tissues between the two groups were performed. TERT expression was negligible in liver tissue from CD-fed mice but highly expressed in liver tissue from EtOH-fed mice (Fig. 3a,b). Moreover, using the double immunofluorescent (IF) analysis, results of Fig. 3c depicts representative colocalization of TERT with macrophage CD68 immunoreactivity in liver tissue. Furthermore, in comparison to CD-fed mice, the mRNA and protein levels of TERT in EtOH-fed mice were distinctly increased in KCs isolated from the liver, respectively (Fig. 3d).

In addition, to address whether TERT has functional role in mouse model of ALD, we analyzed TA in KCs isolated from the liver of EtOH-fed mice or CD-fed mice. Quantification of TA revealed that the development of ALD might be associated with a more than 6-fold increase in TA (Fig. 3e). In concert, these observations demonstrate that TERT is expressed in macrophages of liver tissue, more importantly, EtOH-fed mice have higher levels of TERT than CD-fed mice and telomerase is activated during ALD. Thus, it indicates that up-regulation of TERT may have key roles in the development of ALD, a process driven primarily by macrophages.

### LPS stimulates TERT production in RAW264.7 cells exposed to ethanol

**Figure 4:**
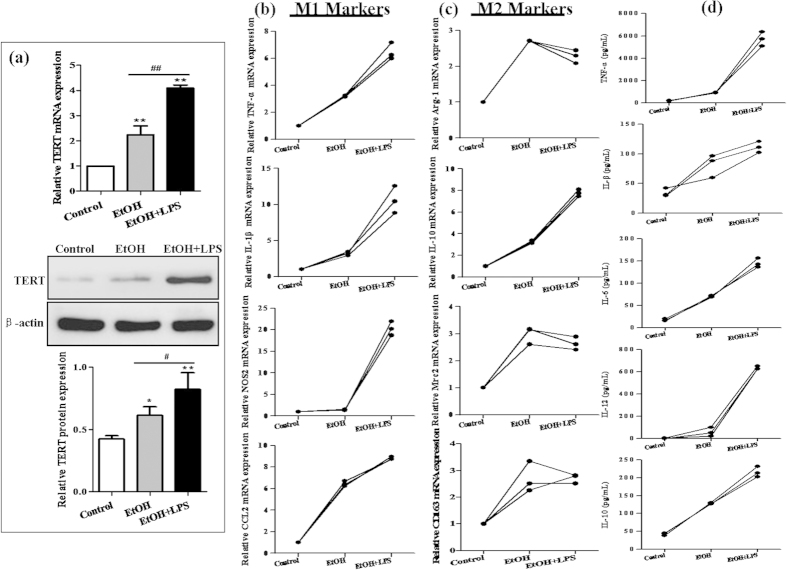
Effect of alcohol on TERT expression *in vitro*. Acute alcohol treatment of RAW 264.7 cells can be achieved with 25 mM EtOH for 24 h. (**a**) TERT mRNA and protein expression in EtOH-stimulated RAW 264.7 cells were analyzed by real-time PCR and western blot. The results are shown as relative expression against control expression without treatment. The values represent means ± SD. *P < 0.05, **P < 0.01 *vs* control. ^#^P<0.05, ^##^P<0.01 *vs* EtOH-treated group. (**b**) Effect of alcohol on M1 macrophage markers (TNF-α, IL-1β, CCL2 and NOS2) in RAW 264.7 cells without or with LPS stimulation. (**c**) Effect of alcohol on M2 macrophage markers (Arg-1, IL-10, Mrc2 and CD163) in RAW 264.7 cells without or with LPS stimulation. (**d**) Effect of alcohol on the production of cytokines including TNF-α, IL-1β, IL-6, IL-12 and IL-10 in RAW 264.7 cells without or with LPS stimulation. The results are shown as line chart.

To test the hypothesis that TERT is induced by ethanol, *in vitro* acute alcohol treatment of macrophages can be achieved with 25 mM EtOH for 24 h^30^. The results of real-time PCR and western blot indicated that ethanol dramatically enhanced the mRNA and protein levels of TERT in RAW264.7 cells. Furthermore, treatment of cells with 1 μg/mL LPS for 24 h caused much more production of TERT in the presence of ethanol (Fig. 4a). These findings suggest that ethanol stimulation can induce TERT expression *in vitro*, and LPS further enhances TERT production in RAW264.7 cells after ethanol exposure.

We further evaluated the expression of macrophage surface phenotypical markers upon ethanol treatment *in vitro*. Real-time PCR results showed that the M1 macrophage markers such as TNF-α, IL-1β and CCL2 were significantly up-regulated in RAW264.7 cells exposed to EtOH, except the NOS2 gene expression. Interestingly, compared with the EtOH group, all of M1 macrophage markers were observably up-regulated in LPS-stimulated RAW 264.7 cells after EtOH exposure (Fig. 4b). Moreover, the mRNA expression of M2 macrophage markers except CD163 was also remarkably increased after stimulation with EtOH, and then their mRNA levels were slightly changed in LPS-treated RAW264.7 cells after EtOH exposure, while the mRNA level of IL-10 was elevated up to 2.4-fold (Fig. 4c). Additionally, the results of ELISA analysis showed that the production of cytokines including TNF-α, IL-1β, IL-6, IL-12 and IL-10 were obviously increased in EtOH-stimulated RAW 264.7 cells, without or with the treatment of LPS (Fig. 4d).

### High expression of TERT in murine M1 macrophages *in vitro*

**Figure 5:**
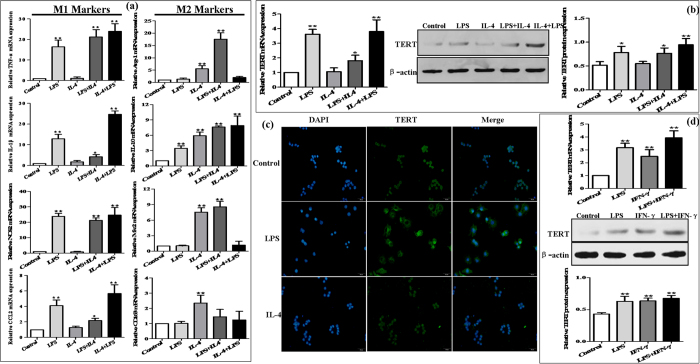
Plastic expression of TERT in murine macrophages. RAW264.7 cells were treated with LPS (1 μg/mL) for 24 h to polarize M1 macrophage phenotype, while treatment with IL-4 (15 ng/mL) for 24 h induced M2 macrophage phenotype. One population into another was transformed by culturing M1 macrophages with IL-4 and M2 macrophages with LPS, respectively. (**a**) The mRNA levels of M1 macrophage markers (TNF-α, IL-1β, CCL2 and NOS2) and M2 macrophage markers (Arg-1, IL-10, Mrc2 and CD163) were analyzed by real-time PCR. (**b**) The plastic expression of TERT in murine macrophage polarization was determined by real-time PCR and western blot. The results are shown as relative expression against control expression without treatment. Data shown are the mean ± SD from 3 independent experiments. *P < 0.05, **P < 0.01 *vs* control. (**c**) The expression of TERT in RAW264.7 macrophages polarization was analyzed by immunofluorescence (IF) assay. Representative views from each group were presented (original magnification, ×20). (**d**) RAW264.7 cells were treated with IFN-γ (10 ng/mL) for 24 h alone or in combination with LPS. The production of TERT was determined by real-time PCR and western blot. The results are shown as relative expression against control expression without treatment. Data shown are the mean ± SD from 3 independent experiments. *P < 0.05, **P < 0.01 *vs* control.

To examine the expression of TERT in murine M1 and M2 macrophages, RAW264.7 cells were treated with 1 μg/mL LPS for 24 h to polarize M1 macrophage phenotype, while treatment with 15 ng/mL IL-4 for 24 h induced M2 macrophage phenotype (Fig. 5a). The results revealed that treatment of RAW264.7 cells with LPS led to up-regulation of TERT mRNA expression within 24 h of stimulation, but IL-4 did not, which was consistent with the protein expression of TERT (Fig. 5b). Furthermore, immunofluorescence (IF) analysis showed that the fluorescence intensity was higher in RAW264.7 cells differentiated with LPS than in cells without stimulation and with IL-4 stimulation, respectively (Fig. 5c). To further account for possible differences in the existing protocols for the *in vitro* activation and differentiation of murine macrophages, the expression levels of TERT in RAW264.7 cells treated with IFN-γ (10 ng/mL) for 24 h alone or in combination with LPS were analyzed. We found that these were similar to those in LPS-treated RAW264.7 cells and the combination of LPS with IFN-γ induced more TERT production (Fig. 5d). Hence, TERT may be up-regulated in murine proinflammatory M1 macrophages, independently of the current protocol for the *in vitro* differentiation.

To further examine whether TERT contributes to the plasticity of macrophage polarization, we tried to transform one population into another by culturing M1 macrophages with IL-4 and M2 macrophages with LPS, respectively. As we expected, treatment of M2 macrophages with LPS resulted in the production of M1 macrophage phenotypic markers such as and TNF-α, IL-1β, NOS2 and CCL2 after stimulation with IL-4 (M2-to-M1, Fig. 5a). Conversely, treatment of M1 macrophages with IL-4 induced the expression of M2 phenotypic markers except CD163 after stimulation with LPS (M1-to-M2, Fig. 5a). M2-to-M1 conversion of macrophages produced more TERT mRNA and protein, whereas M1-to-M2 macrophages had less TERT than those of M1 macrophage, but had still higher level of TERT than those of the control group (Fig. 5b). Taken together, these results indicate that the expression of TERT is rapidly adapted to the different environmental stimuli *in vitro* and TERT may participate in the establishing macrophage plasticity.

### TERT regulates polarization of murine M1 macrophages

**Figure 6:**
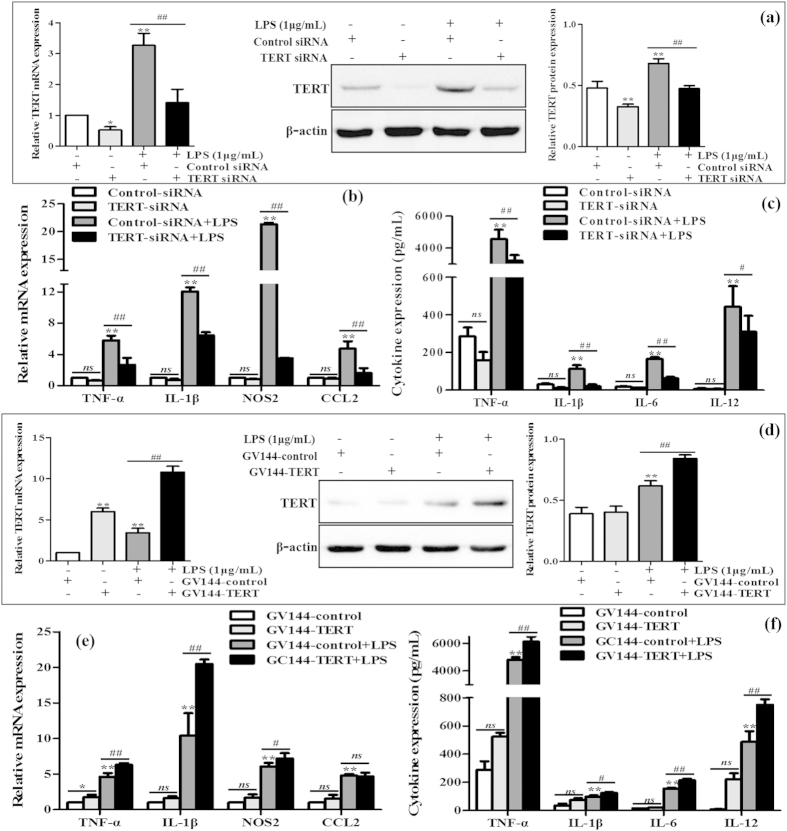
Effect of TERT silencing on murine M1 macrophage polarization. TERT siRNA and GV144-TERT were transiently transfected into LPS-treated RAW264.7 cells, respectively. (**a**) The endogeous TERT levels were detected by real-time PCR and western blot. The results are shown as relative expression against control expression without treatment. (**b**) The mRNA levels of M1 macrophages biomarkers including TNF-α, IL-1β, NOS2 and CCL2 were detected by real-time PCR. The results are shown as relative expression against control expression without treatment. (**c**) The secretion of proinflammatory cytokines including TNF-α, IL-1β, IL-6 and IL-12 were determined by ELISA. (**d**) TERT successful over-expression was verified by real-time PCR and western blot in LPS-stimulated RAW 264.7 cells. The results are shown as relative expression against control expression without treatment. (**e**) The mRNA levels of M1 macrophages biomarkers were detected by real-time PCR. The results are shown as relative expression against control expression without treatment. (**f**) The secretion of proinflammatory cytokines were determined by ELISA. Data shown are the mean ± SD from 3 independent experiments. *P < 0.05, **P < 0.01 *vs* control group. ^#^P < 0.05, ^##^P < 0.01 *vs* LPS-treated group.

To explore the potential role of TERT in M1 macrophage polarization, TERT knockdown and over-expression in LPS-stimulated RAW 264.7 cells were realized by introducing siRNA and plasmids, respectively. Firstly, TERT siRNA was transiently transfected into RAW264.7 cells without or with the treatment of LPS. TERT was successfully silencing endogeous TERT (Fig. 6a). The mRNA levels of M1 macrophages biomarkers including TNF-α, IL-1β, NOS2 and CCL2 were substantially decreased when TERT was blocked (Fig. 6b). In line with the above data, the secretion of proinflammatory cytokines including TNF-α, IL-1β, IL-6 and IL-12 was significantly decreased in TERT-knockdown M1 macrophages compared with that in M1 macrophages transfected with the control-siRNA (Fig. 6c). Conversely, GV144-TERT was used to over-express TERT, and its successful over-expression was verified by the same methods in LPS-stimulated RAW 264.7 cells (Fig. 6d). Cells were harvested after 48 h for real-time PCR and ELISA assays. Over-expression of TERT significantly enhanced the murine macrophage cell populations expressing M1 surface markers, including TNF-α, IL-1β and NOS2, except CCL2, as shown in Fig. 6e. Moreover, we detected the increased levels of TNF-α, IL-1β, IL-6 and IL-12 in LPS-treated macrophages transfected with TERT plasmid compared with vector control (Fig. 6f).

To determine whether TERT influences M2 macrophages polarization as well, TERT plasmid was transiently transfected into IL-4-stimulated RAW264.7 cells for 48 h. The results of real-time PCR and western blot showed that the expression of TERT mRNA and protein was distinctly increased in M2 macrophages transfected with TERT plasmid (Supplementary Fig. S1a). However, we found no differences in the gene expression of M2 surface markers, such as Arg-1, IL-10, Mrc2 and CD163, between the GV144-TERT group and the GV144 group, as shown in Supplementary Fig. S1b. In line with the above data, the production of anti-inflammatory cytokine IL-10 was not increased in TERT over-expression M2 macrophages (Supplementary Fig. S1c).

Collectively, these observations suggest that TERT may regulate murine M1 macrophages polarization, but not directly orient macrophages towards M1 macrophage phenotype *in vitro*. In addition, TERT may unable to induce M2 macrophage polarization.

### TERT regulates M1 macrophages polarization by interacting with p65

**Figure 7:**
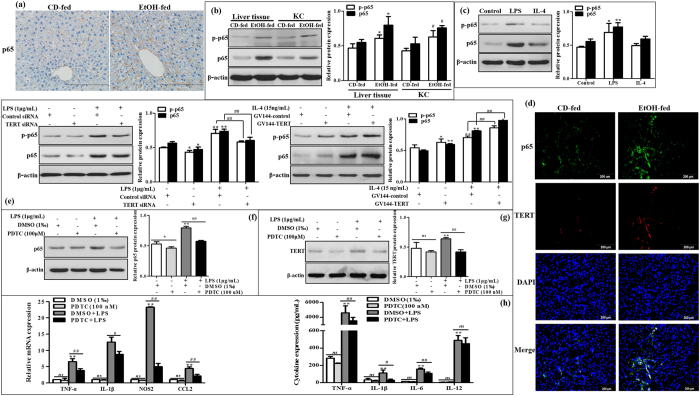
The interaction of TERT with p65 in murine macrophages. (**a**) The expression of p65 in liver tissue was analyzed by IHC staining analysis. Representative views from each group were presented (original magnification, ×40). (**b**) The protein expression and phosphorylation of p65 at Ser 536 were observed in liver tissue and KCs isolated from the liver by western blot. The results are shown as relative expression against control expression without treatment. The values represent means ± SD. (n = 4 in CD-fed group, n=6 in EtOH-fed group) *P < 0.05, **P < 0.01 *vs* liver tissues of CD-fed group. ^#^P < 0.05, ^##^P < 0.01 *vs* KCs of CD-fed group. (**c**) p65 protein expression and phosphorylation were analyzed in total cell lysates of M0, M1 and M2 macrophages by western blot. The results are shown as relative expression against control expression without treatment. Data shown are the mean ± SD from 3 independent experiments. *P < 0.05, **P < 0.01 *vs* control. (**d**) Representative colocalization of TERT with macrophage p65 immunoreactivity in liver tissue by using the double immunofluorescent (IF) analysis. (**e**) Effect of TERT on p65 expression and activation in LPS-stimulated RAW 264.7 cells. The expression of p65 and phosphorylated p65 were determined by western blot. (**f**) Effect of PDTC on p65 expression in LPS-stimulated RAW 264.7 cells. The expression of p65 was determined by western blot. (**g**) The expression of TERT upon treatment with PDTC was determined by western blot in LPS-stimulated RAW 264.7 cells. (**h**) Effect of PDTC on the expression of M1 macrophage biomarkers in LPS-stimulated RAW 264.7 cells. The results are shown as relative expression against control expression without treatment. Data shown are the mean ± SD from 3 independent experiments. *P < 0.05, **P < 0.01 *vs* control. ^#^P < 0.05, ^##^P < 0.01 *vs* LPS-treated group.

To better understand the mechanism by which EtOH increased TERT expression in macrophages *in vivo* and *in vitro* and TERT regulated M1 macrophage polarization, we next identified the signaling cascade which may be involved in ethanol-mediated hepatic proinflammatory response and the effect of TERT on M1 macrophage polarization. Nuclear transcription factor kappa B (NF-κB) is known to regulate numerous genes associated with inflammation and immune cell differentiation and infiltration^31^,^32^. As we expected, activation of the hepatic NF-κB signaling pathway, confirmed by an increase in NF-κB p65 and phosphorylation of p65 at Ser 536, was observed in liver tissue and KCs isolated from the liver of EtOH-fed mice (Fig. 7a,b). Furthermore, the protein expression of p-p65 and p65 was visibly increased in LPS-stimulated RAW 264.7 cells. However, there was no substantial difference in their basal and IL-4-treated RAW 264.7 cells (Fig. 7c). These results suggest that NF-κB signaling pathway is activated in the progression of ALD as well as M1 macrophage differentiation.

Next, to investigate whether TERT influences murine M1 macrophage polarization via NF-κB pathway *in vitro*, the colocalization of p65 and TERT in liver tissue was performed by using the double IF staining (Fig. 7d). Furthermore, western blot analysis showed that knowdown of TERT with siRNA remarkably reduced the protein phosphorylation and expression of p65 in LPS-treated macrophages, while over-expression of TERT observably induced the expression levels of p-p65 and p65 in M1 macrophages (Fig. 7e). Therefore, these data demonstrate that TERT may regulate murine M1 macrophage polarization through NF-κB pathway.

It has previously been found that NF-κB signaling can regulate mouse TERT expression by binding to a site 350 bp upstream from the translational start site^33^. Thus, to further evaluate whether NF-κB pathway is responsible for TERT expression and murine M1 macrophage polarization, Firstly, a chemical inhibitor for NF-κB, PDTC, was administrated to block NF-κB pathway in LPS-stimulated RWA 264.7 cells. The result of western blot showed that p65 protein was significantly suppressed in LPS-stimulated RAW 264.7 cells in the present of 100 μM PDTC (Fig. 7f). Next, we investigated the expression of TERT upon treatment with PDTC. Western blot analysis showed that PDTC obviously inhibited the protein level of TERT in M1 macrophages (Fig. 7g). With respect to the effect of NF-κB on macrophage polarization, the results showed that PDTC could observably suppress the expression of M1 macrophage markers including TNF-α, IL-1β, NOS2, and CCL2, as well as various proinflammatory cytokines TNF-α, IL-1β and IL-6, except IL-12 (Fig. 7h).

In conclusion, these results indicate that TERT acts in a feedback loop with p65 in murine M1 macrophages, which may provide a possible explanation for the ethanol-mediated hepatic proinflammatory response and M1 macrophage polarization *in vitro*.

## Discussion

Accumulating evidence suggests critical roles of TERT in several diseases and pathological conditions, including inflammation, aging and cancer^26^,^27^. Gizard *et al.* demonstrated that macrophages in the shoulder region of human advanced atherosclerotic lesions expressed high levels of TERT and activated telomerase^29^. To the best of our knowledge, there are no data assessing the role of TERT in ALD. In our study, the results showed that TERT levels in liver tissue from EtOH-fed mice were significantly higher than those in CD-fed mice. Harada *et al.* revealed that TR mRNA were detectable in the non-neoplastic hepatocytes, whereas TERT mRNA was only expressed in a few infiltrating mononuclear cells in human chronic viral hepatitis (CVH) and primary biliary cirrhosis (PBC) livers by using *in situ* hybridization (ISH)^34^. Thus, we further found the colocalization of TERT with CD68 in liver tissue. KCs were then isolated from the liver, the results showed that the expression of TERT and TA were remarkably increased in EtOH-fed group compared to CD-fed group. In addition, TERT was also obviously up-regulated in RAW 264.7 cells in response to alcohol exposure, and LPS further enhanced TERT production in alcohol-treated macrophages. In brief, all above findings suggest that up-regulation of TERT and the enhanced TA may play an important role in the development of ALD, a process driven primarily by macrophages.

Activation of KCs to secrete proinflammatory factors is a key event in the initiation of ALD. A growing body of studies have revealed that alcohol-induced endotoxemia can activate hepatic KCs through TLRs and these activated KCs can further contribute to alcohol-induced liver injury in mice^35^,^36^. Our data demonstrated that the number of macrophages in EtOH-fed group was significantly greater than that in the control group. It has previously been found that the liver is populated with different phenotypic subtypes of KCs i.e., M1, M2a, M2b and M2c macrophages in human alcoholic hepatitis (AH)^37^. In our study, several markers of M1 and M2 macrophages were found to be over-expressed robustly in liver tissue and KCs isolated from the liver of EtOH-fed mice, suggesting that both M1 and M2 macrophages are present in mice with ALD. Saha *et al.* also found that chronic ethanol feeding in C57Bl/6 mice for more time (5 weeks) led to the increased expression of M1 macrophage markers (TNF-α, MCP-1, and IL-1β) and M2 macrophage genes (Arg-1, Mrc1, and IL-10)^38^. Furthermore, we further evaluated the effect of ethanol on the expression of macrophage surface phenotypical markers *in vitro*. In line with *in vivo* data, the results showed that the hallmarks of M1 and M2 macrophage activation were significantly induced in RAW264.7 cells exposed to EtOH. In short, our results indicate that alcohol induces a mixed M1/M2 macrophage phenotype.

Given the up-regulation of TERT and the mixed M1/M2 macrophage phenotype in ALD, we further explored the expression profile of TERT in murine M1 and M2 macrophages *in vitro*. The results demonstrated that TERT expression was dramatically induced in LPS-treated macrophages as well as IFN-γ-stimulated macrophages, and more TERT expression was detected in LPS/IFN-γ-stimulated macrophages. These findings are consistent with previous data published by Gizard *et al.*^29^, who noted that TERT expression was induced by LPS, oxLDL, or TNF-α in various macrophages. Meanwhile, M2-to-M1 conversion of macrophages produced more TERT mRNA and protein, whereas M1-to-M2 macrophages had less level of TERT. Taken together, these results indicate that the expression of TERT is induced in murine M1 macrophages *in vitro* and TERT may participate in the regulation of the establishing macrophage plasticity.

The significant increase of TERT expression in KCs isolated from the liver of EtOH-fed mice and M1 macrophages *in vitro* prompted us to explore the possible extra-telomeric roles of TERT in macrophage activation. On one hand, the mRNA levels of TNF-α, IL-1β, NOS2 and CCL2 were substantially reduced when TERT was blocked. Moreover, the secretion of proinflammatory cytokines such as TNF-α, IL-1β, IL-6 and IL-12 was significantly decreased in TERT-knockdown M1 macrophages. On the other hand, over-expression of TERT significantly enhanced the murine macrophage cell populations expressing these M1 surface markers, however, it didn’t affect the hallmarks of M2 macrophage. Collectively, these observations suggest that TERT may regulate murine M1 macrophages polarization, but not influence macrophages towards M2 phenotype *in vitro*. In a word, these findings provide compelling evidence for the non-telomeric functions of TERT, independently of telomere maintenance^39^. The biological significance of these findings implicates novel molecular mechanisms of TERT functions in many essential cellular processes by regulating macrophage polarization besides its well defined function in telomere biology^40^.

NF-κB signaling plays vital roles in LPS-treated macrophage polarization^41^,^42^. Alcohol consumption not only results in alcoholic fatty liver (AFL) but also activates innate immunity via the translocation of bacteria-derived LPS from the gut to the liver. As a stimulus of NF-κB signaling, LPS, can bind LPS-binding protein and then deliver the LPS ligand to CD14 receptor^43^. The LPS-CD14 complex interplays with TLR4 in conjunction with the small extracellular protein MD2 to activate intracellular signaling via NF-κB pathway^44^. In this study, a significant increase in protein expression of p65 and phosphorylation of p65 at Ser 536 was observed in liver tissue and KCs isolated from the liver of EtOH-fed mice as well as in LPS-stimulated RAW 264.7 cells. These data confirm that NF-κB signaling is activated in the progression of ALD and M1 macrophage differentiation. As we all know, stress signals result in IKK kinase complex activation and IκB phosphorylation. Then phosphorylated IκB is gradually ubiquitinated and degraded via the proteosomal pathway. Afterwards, dissociation of IκB can expose the nuclear translocation sites of p65/p50 heterodimers in the promoter region, allowing nuclear translocation and DNA binding^45^,^46^. Many M1 macrophage genes have NF-κB sites in their promoter region, such as NOS2, CCL2 and cyclooxygenase-2 (COX2)^47^. NF-κB can induce the expression of pro-inflammatory cytokines including TNF-α, IL-1β and MCP-1 in response to stress signals^48^. Hence, PDTC as a NF-κB inhibitor could observably suppress the expression of M1 macrophage markers including TNF-α, IL-1β, NOS2, and CCL2, and various proinflammatory cytokines such as TNF-α, IL-1β and IL-6, except IL-12, All above data suggest that chronic alcohol-induced liver injury is related to the activation of NF-κB signaling on hepatic M1 macrophage polarization. Simultaneously, it indicates that IL-12 expression may not solely be mediated by NF-κB pathway, but also through an indirect mechanism.

**Figure 8:**
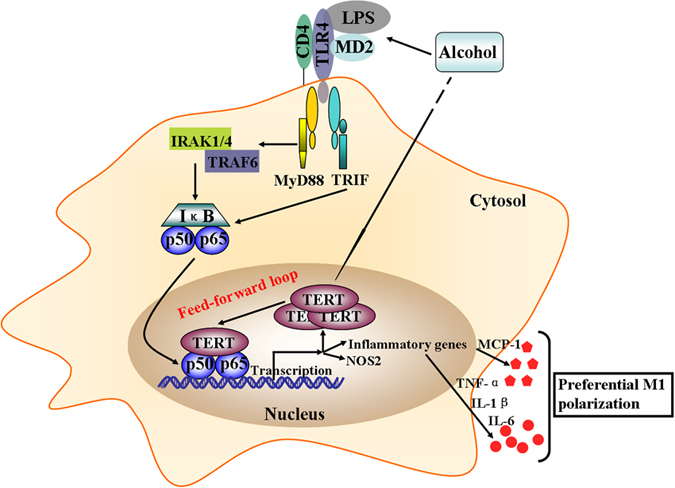
A schematic model of the molecular mechanisms by which TERT acts in a feedback loop with NF-κB pathway to regulate macrophage polarization in a mouse model of chronic binge alcohol-induced ALD. On one hand, Up-regulation of TERT upon the EtOH treatment can induce the expression of M1 macrophage markers including TNF-α, IL-1β, IL-6, NOS2, and CCL2 by interacting with p65, which suggests TERT promotes macrophage polarize towards M1 phenotype via NF-κB signaling. On the other hand, as a stimulus of NF-κB signaling, LPS, can bind LPS-binding protein and then deliver the LPS ligand to CD14 receptor. The LPS-CD14 complex interplays with TLR4 in conjunction with the small extracellular protein MD2 to activate intracellular signaling via NF-κB pathway. Upon activation of TLR4, signaling intermediates IRAK1/4 are recruited to the TLR4 complex via interplay with MyD88 leading to IKK kinase activation.TLR4-induced MyD88- independent (TRIF) signalling results in activation of IKKε and NF-κB as well. Afterwards, NF-κB forms p65/p50 heterodimers in macrophages and binds to the promoter region of TERT and various pro-inflammatory genes to result in gene transcription. In short, this crosstalk mechanism may provide a possible explanation for the ethanol-mediated hepatic proinflammatory response and M1 macrophage polarization *in vitro*.

High throughput analyses of gene expression previously has showed that TERT can regulate almost 300 genes expression, which are involved in cellular processes ranging from cellular signaling to cell proliferation^49^. Furthermore, the assays performed by Ghosh and his associates confirmed that hTERT could directly bind to p65 subunit in the nucleus and regulate the expression of NF-κB-dependent genes such as IL-6 and TNF-α^50^. Based on the results obtained, we further investigated the effect of TERT on NF-κB signaling in macrophage polarization. The double IF staining showed the interaction of p65 and TERT in liver tissue. Moreover, the phosphorylation and protein expression of p65 were dramatically reduced when TERT was silenced in M1 macrophages, while both of them were observably up-regulated in M1 macrophages over-expressing TERT. Therefore, all above data demonstrate that TERT may regulate murine M1 macrophage polarization through targeting NF-κB pathway. In addition, TERT expression was markedly reduced in LPS-stimulated RAW 264.7 cells in response to PDTC. In brief, these results suggest that NF-κB signaling pathway may be responsible for TERT expression in murine M1 macrophages. Importantly, Gizard *et al.* identified a proximal promoter region that conferred TERT transcription, and found a highly conserved NF-κB response element in this region^29^. Given the above, it reveals that there may be a positive feedback loop between TERT and p65 in murine macrophages polarization (Fig. 8). Firstly, TERT regulates murine M1 macrophages activation by increasing the expression of genes such as TNF-α and IL-1β through NF-κB signaling *in vivo* and *in vitro*. Conversely, blockage of NF-κB pathway can reduce TERT expression in LPS-stimulated RAW 264.7 cells. In a word, this mechanism may provide a possible explanation for the ethanol-mediated hepatic proinflammatory response and M1 macrophage polarization *in vitro*. Besides, stimulation of macrophages with other proinflammatory mediators including TNF-α and IL-1β significantly increased the expression of TERT mRNA^29^, which suggests that the downstream signaling molecular of NF-κB pathway may also participate in this feed-forward loop in M1 macrophage activation.

In summary, our study suggests the potential role of TA in non-neoplastic liver. This finding gained support from the work of Invernizzi *et al.*, who also found that since numerous lymphocytes were activated and differentiated continuously in patients with early-stage of primary biliary cirrhosis, TA in peripheral blood mononuclear cells (PBMCs) was higher than that in healthy subjects^51^. Besides, Gizard *et al.* demonstrated that telomerase was significantly activated in the development of atherosclerosis^29^. More importantly, our study strongly indicates the cross-talk of TERT with NF-κB pathway and further provides new insights into non-telomeric functions of telomerase in macrophage polarization. Hopefully, this loop potentially can be used as a novel therapeutic target for ALD. Nowadays, a growing number of studies lead to characterization of various genes and cytoplasmic signaling pathways, which participate in the negative or positive feedback regulation of TERT expression in a majority of cases^40^,^52^. As we all know, TERT has a pleiotropic role in sustained proliferation and survival potentials of various cancer cells. Tumor associated macrophages (TAMs), sharing some characteristics of the M2 macrophage phenotype, have been found to play an essential role in the control of tumor progression by regulating immune suppression and angiogenesis^53^. TERT level in TAMs is currently unknown. Although these findings establish the interaction of TERT with NF-κB signaling in M1 macrophages polarization, it is presently not possible to deduce the reduced expression of TERT in TAMs. Moreover, we cannot exclude the possibility that additional mechanisms might contribute to TERT expression in macrophage activation in TAM. For instance, previous studies revealed that β-catenin could directly regulate TERT expression by interacting with Klf4, which is a core component of the pluripotency transcriptional network^54^. It has also been found that KLF4 can cooperate with STAT6 to induce M2 genes including Arg1 in the stimulation of IL-4^55^. In light of the above hypothesis, TERT may inversely enhance their degree of M2 polarization in cancer cells. It suggests that dynamic changes in TERT expression may contribute to an innate mechanism in which macrophages are effectively respond to various environmental cues.

## Materials and Methods

### Animal, mouse model of ALD

All the animal experimental procedures were approved by the Ethics Committees of Anhui Medical University, and were reviewed and performed in accordance with the Guideline of Animal Care and Use Committee of Anhui Medical University. 8-week-old male C57BL/6J mice from the Experimental Animal Center of Anhui Medical University were used for ALD model. For all experiments, mice were divided randomly into CD-fed group and EtOH-fed group. ALD was generated by using the National Institute on Alcohol Abuse and Alcoholism (NIAAA) recommended Lieber-DeCarli (LD) liquid diet plus alcohol gavage^56^. The diet was purchased from TROPHIC Animal Feed High-Tech Co. Ltd (Hai’an, Jiangsu, China). Modeling process includes a liquid diet adaptation period (5 days), modeling (10 days), gavage (1 time) and specimens (1 day), a total of 16 days. The EtOH-fed mice were fed ethanol (5% v/v) LD liquid diets ad libitum for 10 days plus a single binge ethanol administration (5 g/kg, body weight, 20% ethanol) by gavage, whereas the CD-fed mice were fed and gavaged with isocaloric maltose-dextrin. Both diets were prepared fresh daily. 9 h after the last gavage alcohol injection, mice were anesthetized, and blood and liver tissues were harvested for the further analysis. Plasma was stored at −80 °C. Portions of liver tissue were frozen immediately in liquid nitrogen, whereas others were fixed in 10% neutral-buffered formalin for H&E staining and Oil red O staining, or embedded in frozen specimen medium (TissueTek OCT compound; Sakura Finetek, Torrance, CA).

### Isolation of liver kupffer cells (KCs)

Isolated liver KCs were collected by *in situ* collagenase (type IV; V900893, Sigma-Aldrich, St. Louis, USA) perfusion and differential centrifugation on OptiPrep (Sigma-Aldrich, St. Louis, USA) density gradient as described previously^57^,^58^. Briefly, a 20-G catheter was put through mouse the portal vein, and the inferior vena cava cut. The liver was per fused with PB, followed by a digestion buffer [1 × PBC, supplemented with collagenase, Pronase E, and 4.76 mM CaCl_2_]. After digestion, the liver was disrupted in BSA solution (1 × PBC, supplemented with 0.5% FBS). Single cells were passed through a 200-μm cell strainer, and cells were fractionated using 25% Percol and 50% Percol (Sigma-Aldrich, St. Louis, USA). The intercushion fraction was washed and then adhered to plastic in medium. Furthermore, KCs adhered to the plastic, whereas the nonadherent fraction was washed off in DMEM with 10% FBS. KCs from 2 mice were pooled, given the limited number of KCs available from each animal for RNA and protein isolation.

### Cell culture and cell treatment

RAW264.7 (No. TCM13) cells were purchased from the Type Culture Collection of the Chinese Academy of Sciences (Shanghai, China). Cells were maintained in Dulbecco’s modified Eagle’s medium (DMEM, Gibco, USA) supplemented with 10% fetal bovine serum (FBS, Millipore, USA), 100 U/mL penicillin, 100 mg/mL streptomycin and incubated at 37 °C at an atmosphere of 5% CO2. RAW264.7 cells were cultured in DMEM media with lipopolysaccharides (LPS, 1 μg/mL, No.L2880, Sigma-Aldrich, St. Louis, MO, USA) for 24 h to generate M1 macrophages, while cells were cultured in DMEM media with IL-4 (15 ng/mL, No. 214-14, PeproTech, Rocky Hill, USA) for 24 h to generate M2 macrophages.

### Flow cytometry analysis

The isolated liver KCs were resuspended at a concentration of ~10^6^ cells/100 μL in FACS staining buffer containing anti-CD45, anti-F4/80 and anti-CD11b. FITC-conjugated anti-mouse F4/80 (Clone BM8, No.11-4801) was obtained from eBioscience (San Diego, CA, USA). PE/Cy7-conjugated anti-mouse CD45 (Clone 30-F11, No.103114) and APC/Cy7- conjugated anti-mouse CD11b (Clone M1/70, No.101226) were purchased from Biolegend (San Diego, CA, USA). CD45^+^ cells were gated to exclude endothelial cells, stellate cells, and residue hepatocytes, and the percentage of F4/80^+^ and CD11b^+^ was determined from the CD45^+^ population. For the negative control, cells were stained with isotype-matched control antibodies. Cells were acquired on a BD LSR II instrument (BD Biosciences, San Jose, CA, USA), and the data was analyzed by FlowJo software (Tree Star, Inc., Ashland, OR, USA).

### Serum levels of TG and TCH analysis and ALT/AST activity assay

Alanine aminotransferases (ALT, C009-2) assay kit, aspartate aminotransferases (AST, C010-2) assay kit, triglyceride (TG, A110-1) and total cholesterol (TCH, A111-1) assay kits were from Jiancheng Biology Institution PeproTech (Nanjing, Jiangsu, China).The serum levels of TG and TCH in mice with chronic binge alcohol-induced ALD were analyzed using TG and TCH assay kits according to the protocols. ALT and AST levels in serum from C57BL/6J mice with ALD were analyzed by using ALT and AST activity assay kits according to the protocols recommended by the manufacturer. The absorbance at 510 nm was obtained with a micro-plate reader model 680 (Bio-Rad Laboratories, Hercules, CA, USA).

### Total RNA extraction and quantitative real-time PCR

Total RNA was extracted from mouse liver tissues, KCs and RAW264.7 cells using TRIzol reagents (Invitrogen, USA), and the first-strand cDNA was synthesized using Thermoscript RT-PCR synthesis kit (Fermentas, USA) according to the manufacturer’s instructions. Real-time quantitative PCR analyses for mRNA of TERT, TNF-α, IL-1β, NOS2, CCL2, Arg-1, IL-10, Mrc2, CD163, and β-actin were performed by using Thermoscript RT-qPCR kits (Fermentas, USA) in an ABI Prizm step-one plus real-time PCR System (Applied Biosystems, USA). The mRNA level of β-actin was used as an internal control. Primer sequences were listed in Supplementary Table S1. Relative expression levels were calculated according to the standard 2^−ΔΔCt^ method. All experiments were performed in triplicate and repeated at least three times.

### ELISA assay

The TNF-α, IL-1β, IL-6, IL-12, MCP-1, IL-10 and TGF-β concentrations in the cell supernatants and mouse serum were determined using RayBio® Mouse ELISA Kit (RayBiotech, Inc., Atlanta, GA, USA). Assays were performed using the protocols recommended by the manufacturer. Prepare all reagents, samples and standards as instructed. Add 100 μl standard or sample to each well and incubate 2.5 h at room temperature or over night at 4 °C. Then add 100 μl prepared biotin antibody to each well and incubate 1 h at room temperature. Add 100 μl prepared streptavidin solution and incubate 45 min at room temperature. And then add 100 μl TMB one-step substrate reagent to each well and incubate 30 min at room temperature. Add 50 μl stop solution to each well and the absorbance at 450 nm of each well was immediately measured by using a Thermomax microplate reader (bio-tekEL, U.S.A.). All experiments were performed in triplicate.

### Immunofluorescence (IF) staining

To determine the location and expression of TERT *in vitro*, FITC-conjugated anti-TERT antibody (1:100) was used in IF staining. Cells were mounted with SlowFade Gold antifade reagent with DAPI (Sigma, MO, USA) and images were taken using fluorescence microscopy. TERT was shown as green fluorescence and cell nuclei as blue fluorescence. Tissues from mice livers were fixed with 4% paraformaldehyde and were permeabilized with 0.2% Triton X-100 in 1% bovine serum albumin (BSA) for 10 min, blocked with 5% BSA for one hour at room temperature. To investigate the colocalization of TERT and CD68, as well as TERT and p65, Cy3-conjugated anti-TERT antibody (1:100) in combination with FITC-conjugated anti-CD68 antibody (1:50), Cy3-conjugated anti-TERT antibody (1:100) in combination with FITC-conjugated anti- p65 antibody (1:400) were used in the hybridization assays. Cells were mounted with SlowFade Gold antifade reagent with DAPI and images were taken using inversion fluorescence microscopy. TERT was shown as redfluorescence, and p65 and CD68 as greenfluorescence, respectively.

### Immunohistochemistry

Liver tissues were fixed in 10% neutral buffered formalin solution, embedded in paraffin, and stained for routine histology. Slides were dewaxed in xylene and dehydrated in alcohol, and antigen retrieval was achieved by microwaving in citric saline for 15 min. The sections were deparaffinized and treated with 0.3% hydrogen peroxide for 15 min to block endogenous peroxidase activity. The sections were further blocked by 2% bovine serum albumin followed by incubation with primary antibody against CD68 (1:200, BA3638, Wuhan, Hubei, China), TERT (1:50) and p65 (1:800) for 16 h at 4 °C. After rinsing, the sections were incubated with biotinylated secondary antibody for 60 min at room temperature. CD68, TERT and p65 expression was visualized by 3,3′-diaminobenzidine tetrahydrochloride (DAB) staining. The sections were counter stained with Mayer’s hematoxylin for 30 s, dehydrated, and CD68, TERT and p65 positive areas within the alcoholic liver injury region were then observed.

### Western blot analysis

Mouse liver tissues, KCs and RAW264.7 cells were lysed with RIPA lysis buffer (Beyotime, Shanghai, China). Whole extracts were prepared, and protein concentration was detected using a BCA protein assay kit (Beyotime, Shanghai, China). Total protein (30 or 50 mg) from samples were separated by SDS-PAGE and blotted onto a PVDF membrane (Millipore, Billerica, MA, USA). After blockade of nonspecific protein binding, nitrocellulose blots were incubated for 1 h with primary antibodies diluted in TBS/Tween-20 (0.075%) containing 3% Marvel. Mouse monoclonal antibody recognizing TERT (NB100-317, Novus Biologicals, Littleton, CO, USA) was used 1:500 as was anti-β-actin. Rabbit monoclonal antibody recognizing p65 (#8242, Cell Signaling Tech, Danvers, MA, USA) was used 1:1000 as was anti-p-p65 (#3033, Cell Signaling). Horseradish peroxidase conjugated anti-rabbit and anti-mouse antibodies were used as secondary antibody. After extensive washing in TBS/Tween-20, the blots were processed with distilled water for detection of antigen using the enhanced chemiluminescence system. Proteins were visualized with ECL-chemiluminescent kit (ECL-plus, Thermo Scientific). All experiments were repeated three times.

### Analysis of Telomerase Activity (TA)

TA of KCs was measured by the telomeric repeat amplification protocol (TRAP) method using a quantitative Telomerase Detection Kit (Allied Biotech, Inc.) according to the manufacturer’s instructions. PCRs were performed by using whole-cell extract containing 0.4 μg of protein for 36 cycles of denaturation/ annealing/extension steps.

### RNA interference (RNAi)

RNAi was performed by using Lipofectamine^®^ 3000 (L3000008, Invitrogen, Carlsbad, CA) according to the manufacturer’s protocol. Small interfering RNA (siRNA) oligonucleotides against TERT gene were synthesized by GenePharma (Shanghai, China). A negative scrambled siRNA (GenePharma, Shanghai, China) was used in parallel. RAW264.7 cells were cultured in serum-free DMEM for 12 h and then subjected to reverse transfection with siRNA-mTERT or the scrambled sequences in Opti-MEM (Gibco, USA). The culture medium was changed 6 h after transfection, and LPS (1 μg/mL) was added. The siRNA sequences were as follows: siRNA-TERT (mouse), 5′-CAGAUCAAGAGCAGUAGUCTT-3′ (sense) and 5′-GACUACUGC- UCUUGA UCUGTT-3′ (antisense). Transfection was allowed to proceed for various times and cells were processed for different assays. The siRNA transfection efficiency of Lipofectamine^®^ 3000 in cells was determined by the BLOCK-iT Alexa FluorR Red Fluorescent Oligo protocol (Invitrogen, USA). All experiments were repeated three times.

### Recombinant mouse TERT plasmid (pTERT) construction

pTERT was purchased from Genechem (Shanghai, China) and extracted by using Plasmid Extraction Kit (Tiangen Biotech, Beijing, China). Ectopic expression of mTERT was achieved by using GV144-TERT transfection and empty vector GV144 (CMV-EGFP-MCS-SV40-Neomycin) was used as a control. The constructed plasmid was transfected into RAW264.7 cells and observed under fluorescence microscopy assays. All experiments were performed in triplicate and repeated at least three times.

### Statistical analysis

Data are presented as means ± SD and were analyzed using SPSS_16.0 software. Statistical significances were determined by one-way ANOVA with the post-hoc Dunnett’s test. In all cases, values of P < 0.05 were considered to be statistically significant.

## Additional Information

**How to cite this article**: Wu, X.- *et al.* Telomerase reverse transcriptase acts in a feedback loop with NF-κB pathway to regulate macrophage polarization in alcoholic liver disease. *Sci. Rep.* **6**, 18685; doi: 10.1038/srep18685 (2016).

## Supplementary Material

Supplementary Information
