# Supplementary material for: Telomerase reverse transcriptase acts in a feedback loop with NF-κB pathway to regulate macrophage polarization in alcoholic liver disease
Source: Sci Rep. 2016 Jan 4;6:18685. doi: 10.1038/srep18685 (PMC4698632; doi:10.1038/srep18685)

Supplementary information

Telomerase reverse transcriptase acts in a feedback loop with NF-κB pathway to regulate macrophage polarization in alcoholic liver disease

Xiao-qin Wu, Yang Yang, Wan-xia Li, Ya-hui Cheng, Xiao-feng Li, Cheng Huang, Xiao-ming Meng, Bao-ming Wu, Xin-hua Liu, Lei Zhang, Xiong-wen Lv & Jun Li*

Table S1. Nucleotide sequence of primers used for real time-PCR.

Gene	Accession no.	Forward	Reverse
TERT	NM_009354.1	5'-GGCTCTTCTTCTACCGTAAG-3'	5'-TGATGCCTGACCTCCTCTTG-3'
TNF-α	NM_001278601.1	5'-CACCACCATCAAGGACTCAA-3'	5'-AGGCAACCTGACCACTCTCC-3'
IL-1β	NM_008361.3	5'-CTTTGAAGTTGACGGACCC-3'	5'-TGAGTGATACTGCCTGCCTG-3'
NOS2	NM_010927.3	5'-AATCTTGGAGCGAGTTGTGG-3′	5'-CAGGAAGTAGGTGAGGGCTTG-3′
CCL2	NM_011333.3	5'-GGGCCTGCTGTTCACAGTT-3′	5'-CCAGCCTACTCATTGGGAT-3′
Arg-1	NM_007482.3	5'-CTCCAAGCCAAAGTCCTTAGAG-3′	5'-AGGAGCTGTCATTAGGGACATC-3′
Mrc2	NM_008626.3	5'-TGCAAGCAATGCATCCAAGCCT-3′	5'-ACGGCTTTCCGTGTGAGTTT-3′
IL-10	NM_010548.2	5'-GCCTTGCAGAAAAGAGAGCT-3′	5'-AAAGAAAGTCTTCACCTGGC-3′
CD163	NM_001170395.1	5'-TGGGTGGGGAAAGCATAACT-3′	5'-AAGTTGTCGTCACACACCGT-3′
GAPDH	NM_001289726.1	5'-GGACCTCATGGCCTACATGG-3′	5'-TAGGGCCTCTCTTGCTCAGT-3′

Supplementary figure legends

Figure S1. Effect of TERT over-expression on murine M2 macrophage polarization. GV144-TERT was transiently transfected into RAW264.7 cells without and with IL-4 stimulation. (**a**) TERT successful over-expression was verified by real-time PCR and western blot in IL-4-stimulated RAW 264.7 cells. The results are shown as relative expression against control expression without treatment. (**b**) The mRNA levels of M2 macrophages biomarkers including Arg-1, IL-10, Mrc2 and CD163 were detected by real-time PCR. The results are shown as relative expression against control expression without treatment. (**c**) The secretion of proinflammatory cytokine IL-10 was determined by ELISA. Data shown are the mean ± SD from 3 independent experiments. *P<0.05, **P<0.01 *vs* control group. #P<0.05, ##P<0.01 *vs* IL-4-treated group.

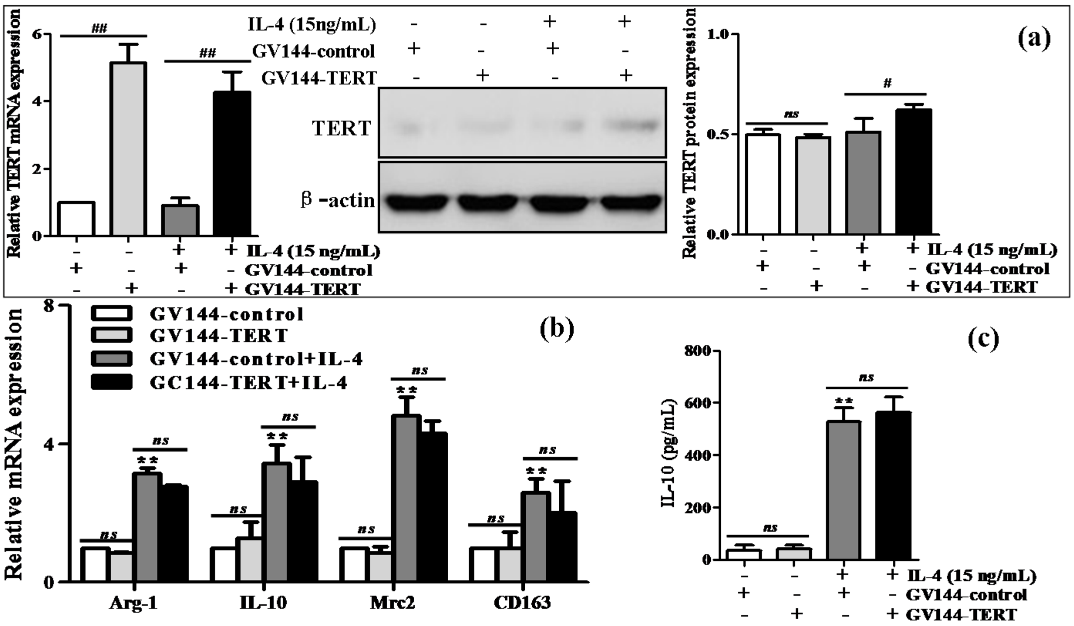

Supplement: Supplementary Information [file srep18685-s1.pdf]
